# Hidden Causes of Variation in Offspring Reproductive Value: Negative Effects of Maternal Breeding Age on Offspring Telomere Length Persist Undiminished Across Multiple Generations

**DOI:** 10.1111/ele.70043

**Published:** 2024-12-31

**Authors:** Valeria Marasco, Winnie Boner, Kate Griffiths, Shirley Raveh, Pat Monaghan

**Affiliations:** ^1^ Department of Interdisciplinary Life Sciences, Research Institute of Wildlife Ecology University of Veterinary Medicine Vienna Vienna Austria; ^2^ School of Biodiversity, One Health and Veterinary Medicine University of Glasgow Glasgow UK

## Abstract

Offspring of older breeders frequently show reduced longevity, which has been linked to shorter offspring telomere length. It is currently unknown whether such telomere reduction persists beyond a single generation, as would be the case if germline transmission is involved. In a within‐grandmother, multi‐generational study using zebra finches, we show that the shorter telomeres observed in F1 offspring of older mothers are still present in the F2 generation even when the breeding age of their F1 mothers is young. The effect was substantial: 43% shorter telomeres in grandoffspring from the ‘grandmother old at breeding’ line compared with those from the ‘grandmother young at breeding’ line. Shorter telomeres at fledging in this species are associated with a reduction in lifespan. Our data demonstrate the need to look beyond a single generation to explain inter‐individual variation in ageing rates and thereby variation in optimal allocation of age‐specific reproductive effort.

## Graphical abstract

In a within‐grandmother, multi‐generational study using zebra finches, we show that the shorter telomeres observed in fledgling offspring (F1) of older mothers (F0) persist into a further generation (F2). These data are in line with the predictions based on a germline‐transmitted effect of maternal age on offspring telomere length due to oocyte deterioration in older mothers. Our data demonstrate the need to look beyond a single generation to explain inter‐individual variation in ageing rates and thereby variation in optimal allocation of age‐specific reproductive effort.

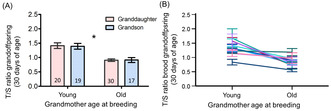

## 1 Introduction

Across a wide diversity of iteroparous species, reproductive performance declines with age, largely due to senescence (Lemaître and Gaillard 2017; Monaghan and Ivimey‐Cook 2023). However, variation in the fitness benefits resulting from offspring produced at different parental ages has been neglected in life‐history theory, despite its importance in shaping investment trade‐offs and thereby the evolution of optimal scheduling of reproductive effort across the life course (McNamara and Houston 1996). A widely documented phenomenon in a large variety of vertebrate and invertebrate species is the ‘Lansing effect’, a decrease in offspring lifespan with increased parental age at breeding (Lansing 1947; Monaghan, Maklakov, and Metcalfe 2020). This could reduce the reproductive value of offspring produced at older ages. For such parental age effects to be important in an eco‐evolutionary context, they need to persist across multiple generations. It is important therefore to examine whether parental age effects on offspring can still be demonstrated in subsequent generations who have not themselves experienced the environmental trigger (Perez and Lehner 2019), in this case having an old parent.

Examining effects of parental age on offspring longevity is difficult, due to factors such as the selective disappearance of phenotypes (i.e. earlier mortality of poor‐quality parents causing changes in the phenotypic composition of age groups (Nussey et al. 2008)). In experiments with Neriid flies, Wylde et al. (2019) found an effect of both grandmother and grandfather ages on baseline offspring mortality but could not rule out potentially confounding effects of differential survival of parent phenotypes into old age. To identify multi‐generational effects of parental age, a within‐individual approach is needed in which age at breeding of parents and grandparents can be controlled for at least three generations, with environmental conditions being held constant and controlled (Burton and Metcalfe 2014; Perez and Lehner 2019). While effects of parental age on offspring can operate through either the father or the mother, effects of maternal age at breeding, which is the focus of this paper, are likely to be stronger due to the higher maternal per capita investment in offspring, the potentially long storage time of oocytes which is associated with oocyte deterioration (particularly in birds and mammals (Monaghan and Metcalfe 2019)), and maternal influence on offspring development.

Adverse effects of maternal age on offspring longevity could occur through a number of different routes (Monaghan, Maklakov, and Metcalfe 2020). Here, we examine whether multi‐generational effects on offspring telomere dynamics can be detected. Reduction in the number of repeats of the telomeric sequence at chromosome ends is considered a key hallmark of ageing (López‐Otín et al. 2023) and is associated with increased frailty, faster ageing and reduced lifespan in multiple taxa in the field and laboratory (Shay and Wright 2019). Measuring telomere length as an indicator of biological state is particularly useful for ecologists since samples can often be obtained relatively non‐invasively. This is especially so for birds, in which a consistent DNA sample of known cell composition can be obtained from red blood cells (rbc). Telomere length reduction can occur through multiple cellular pathways, and shorter telomere length reduces the replicative potential of cells, increasing the rate of cellular and eventually organism senescence (Shay and Wright 2019). By tracking telomere length across the lifespan of a cohort of zebra finches ( *Taeniopygia guttata* ), we have previously shown that red blood cell telomere length at fledging is a strong predictor of subsequent lifespan (Heidinger et al. 2012). We have also found that, even in benign and consistent conditions in captivity, in the absence of mate choice, both maternal and paternal ages are associated with reduced offspring lifespan (Noguera, Metcalfe, and Monaghan 2018). To identify mechanisms underlying this, we previously compared rbc telomere length in the first generation (F1) offspring of a group of mothers (F0) who bred in young, and then again in old age, always with young males randomly allocated by the experimenters. We found that the F1 sons and daughters produced when the mothers were old had rbc telomeres shorter on average by 36% at fledging compared with those F1 offspring these same mothers had produced as young breeders (Marasco et al. 2019). This could arise due to age‐related deterioration of parental care capability in mothers adversely affecting offspring telomere loss during development; in this case, it would not persist beyond a single generation, and thus not be present when the F1 offspring breed at a young age. It is also possible that the parental care capacity of the F1 offspring is adversely affected by having an old mother, even when they are young breeders, and that poor rearing conditions reduce telomere length in the F2 generation by this route. However, given that the birds were breeding in captivity, in benign and protected environmental conditions, with ad libitum food, and that parental care was also provided by the young fathers, this seems an unlikely route. Nonetheless, we checked for any evidence of poor condition or breeding performance in the young F1 breeding females and whether their offspring showed normal growth. Alternatively, offspring of an old mother might inherit shorter telomeres due to ageing of their mother's oocytes, in which case the effect could be transmitted to both the soma and the germline of their F1 offspring; in this case, a grandmother effect would be evident in the F2 offspring, even when the F1 offspring breed at a young age (Monaghan and Metcalfe 2019).

To test this, we bred the F1 daughters that had been produced by the same 11 mothers (F0) as both young and old breeders. These F1 daughters were raised in our standardised laboratory conditions. They were paired when they were young adults, with unrelated young adult males, to give the F2 generation. Such a within‐grandmother design excludes any selective disappearance effects in the F0 generation, young fathers allocated by the experimenters exclude any paternal age or mate choice effects, and breeding the F1 offspring at a young age excludes any direct effects of parental somatic ageing. All breeding events took place in the same environmental conditions, with ad lib food and temperature control, hence also excluding any other direct environmental effects.

## 2 Materials and Methods

### 2.1 General Husbandry

All procedures were carried out under the UK Home Office (HO) Project Licence 60/4109 held at the University of Glasgow. The 11 grandmothers (F0 females) in this study were produced from the breeding colony at the University of Glasgow and their offspring were reared in this facility. The standard husbandry regime involves an ad libitum supply of mixed seeds—common millet, yellow millet and canary seed in a ratio of 3:1:1 (Johnson and Jeff, UK)—cuttlebone, weekly protein supplement and ad libitum water. Photoperiod was maintained at 14 h:10 h light: dark cycle and the temperature between 20°C and 24°C. When breeding, pairs were housed in an HO‐approved breeding cage (60 × 50 × 50 cm) equipped with an external nest box and nest material (coconut fibres, Haiths Ltd.).

### 2.2 F0, F1 and F2 Birds

The F0 birds in this study formed part of a larger study of F1 maternal effects (Marasco et al. 2018) and comprise the subset of 11 F0 females in that study who bred both in young (6 months old) and in old ages (42 months old) and produced daughters in the F1 generation. These F0 females had been produced from young mothers (mean age ± se: 16.3 ± 1.5 months) in our stock population. The F0 females bred with unrelated but otherwise randomly selected males kept in the standard conditions that were relatively young (mean age ± se: 11.2 ± 1.5 months) to exclude any confounding effects of father age on the telomere length of the offspring produced. As part of the larger F1 study, some of the F0 females were exposed to an environmental stressor (unpredictable food regime that increased plasma corticosterone when food was absent, without causing any food restriction (Marasco et al. 2018), which was the case for 8 out of the 11 F0 females in this study). There was no effect of this manipulation on the telomere length of their F1 daughters in either the main study (Marasco et al. 2019) or the subset used here (General Linear Mixed Model: maternal environment, *p* = 0.5). Maternal age, on the other hand, had a substantial effect on daughter telomere length, with rbc telomere length in the daughters produced when the F0 females were old being substantially reduced (Marasco et al. 2019). The F1 daughters that these 11 F0 females produced as young and as old breeders (*n* = 11 and 12 daughters, respectively) were housed in the same control benign conditions until they bred as young adults (mean age ± se: 230 ± 8 days) with young unrelated males kept in similar conditions (mean age ± se: 426 ± 22 days) to produce the grandoffspring (F2). We recorded the body mass of the F1 females at breeding, their clutch sizes, number and body mass of young (F2) at fledging (~30 days of age; age estimated from when the first chick hatched within each clutch) using an electronic balance (0.1 g) prior to blood sampling (see below).

### 2.3 Measurements of Telomere Length

F0, F1 and F2 birds were blood sampled at fledging. Small blood samples (up to 70 μL) were collected by venipuncture of the alar vein and immediately placed on ice. Hatching order within each nest was also recorded since this can influence telomere length (Noguera et al. 2016). Within 4 h of collection, the blood samples were spun to separate plasma from red blood cells (10 min at 0.4g, 4°C), and the latter were stored at −80°C until later analysis. Telomere length was measured in rbc of 30‐day‐old F0 grandmothers, the F1 daughters they produced in young and in old age, and in the F2 offspring their F1 daughters produced as young breeders.

In the telomere lab at the University of Glasgow, DNA from rbc was extracted using commercial kits and following the manufacture's protocol (Macherey‐Nagel, USA). Relative telomere length (RTL) was quantified in the extracted DNA by using qPCR following the same protocol as in Marasco et al. (2019), and in accordance with the MIQE guidelines (Bustin et al. 2009). Briefly, the relative telomere length of each sample was measured by determining the ratio (T:S) of telomere repeat copy number (T) to a single‐copy control gene (S), relative to the same DNA reference sample run on each plate. Glyceraldehyde‐3‐phosphate dehydrogenase (GAPDH) was used as the single‐copy control gene. The telomere and GAPDH reactions were carried out on separate plates, and in both reactions the number of PCR cycles (Ct) required for the products to accumulate enough fluorescent signal to cross a threshold was determined. Reaction efficiencies were within the acceptable range (i.e. 100% ± 10%). All samples fell within the bounds of the standard curve run on every plate (6 standard dilutions, from 40 to 125 ng of DNA). Each plate contained a standard curve, and all standards and samples were run in triplicate. The intra‐plate coefficient of variation for the telomere and GAPDH assays for the raw Ct values were 0.69% and 0.98%, respectively; the inter‐plate coefficient of variation calculated using the same standard dilutions and the same reference sample that were run across each plate for both the telomere and GAPDH assays were 1.7% and 1.0%, respectively. We lack telomere length data from one F1 female (from the ‘F0 old at breeding’ line) due to a missing blood sample and from one F2 offspring (from the ‘F0 young at breeding’ line) due to poor‐quality DNA.

### 2.4 Statistics

Data were analysed in R (version 4.2.2; R core team, 2022) using General Linear Model (GLM) or Generalised Linear Mixed Models (GLMM) with a Gaussian distribution [‘lme4’, ‘lmerTest’‐ (Bates et al. 2015)]. A GLMM was performed to analyse whether grandmaternal (F0) age at breeding influenced telomere length (ln‐transformed to improve normalisation of model residuals) of the grandoffspring (F2) at fledging. We included grandmaternal age at breeding (young or old), grandoffspring sex (male or female), grandoffspring body mass at sampling (fledging), hatching position and clutch size from which F2 birds originated as fixed predictors. As telomere length in the F2 offspring was positively correlated with telomere length of their mothers (F1) at fledging (the latter measurements were available in 22 out of 23 mothers; *r* = 0.47, *p* < 0.001), we also tested whether variation in grandoffspring fledging telomere length was associated with variation in maternal fledging telomere length either as main effect or in its interaction with grandmaternal age (collinearity among the explanatory variables was tested using the VIF function and was < 2, which is considered low); both these two factors were not significant (*p* ≥ 0.1) and were thus removed from the final telomere length model output. Separate GLM or GLMM was also performed to examine any potential effect of the age of F0 females at reproduction (young or old) on the body mass at pairing of their daughters (F1), and their subsequent breeding performance (clutch sizes, number of offspring (F2) reared until fledging and their fledging mass). Due to the limited variation in the age of the F1 daughters and their mates at reproduction (mean age ± se: 230 ± 8 days and 426 ± 22 days, respectively), we did not include either maternal or paternal age at breeding as predictors in the analyses of F2 grandoffspring fledging mass or fledging telomere length. Finally, we performed two GLMMs to examine the change in average 30‐day telomere length of females spanning the three generations, separating the F1 and F2 generations resulting from the young‐ and the old‐F0 grandmother at breeding line. We carried out pair‐wise post hoc contrasts across generations for significant fixed factors using the R package ‘emmeans’ with Tukey's *p*‐values adjustment (Lenth et al. 2023). As appropriate, in all GLMMs, we entered the identity of grandmothers (F0) and/or their mothers (F1) as random factors (intercept) to control for possible pseudoreplication due to F2 birds sharing the same grandmothers (F0) and/or mothers (F1). Figures were generated using GraphPad Prism 5.00 and SPSS 29.0.1.0.

## 3 Results

**FIGURE 1:**
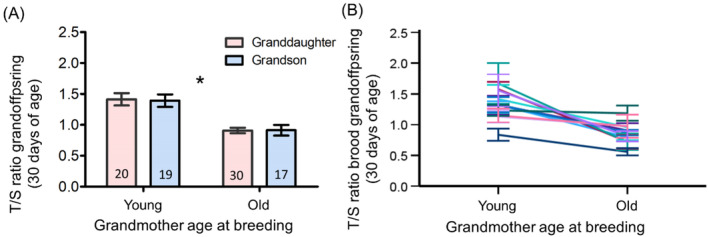
Grandoffspring (F2) from the old ‘grandmother (F0) at breeder’ line are ‘older’ than their chronological age. (A) Thirty‐day telomere length (mean ± se) of the F2 offspring that were produced by the F1 daughters of the same 11 F0 females who bred in both young and old ages; Numbers in (A) indicate F2 offspring sample sizes separately by sex (there were no differences in the sex ratio between the grandmothers´ two breeding events: Chi‐squared test = 0.91, df = 1, *p* = 0.34); * denotes *p* < 0.0001. (B) Mean telomere length for each of the broods (± se) connected by F0 grandmother identity.

**FIGURE 2:**
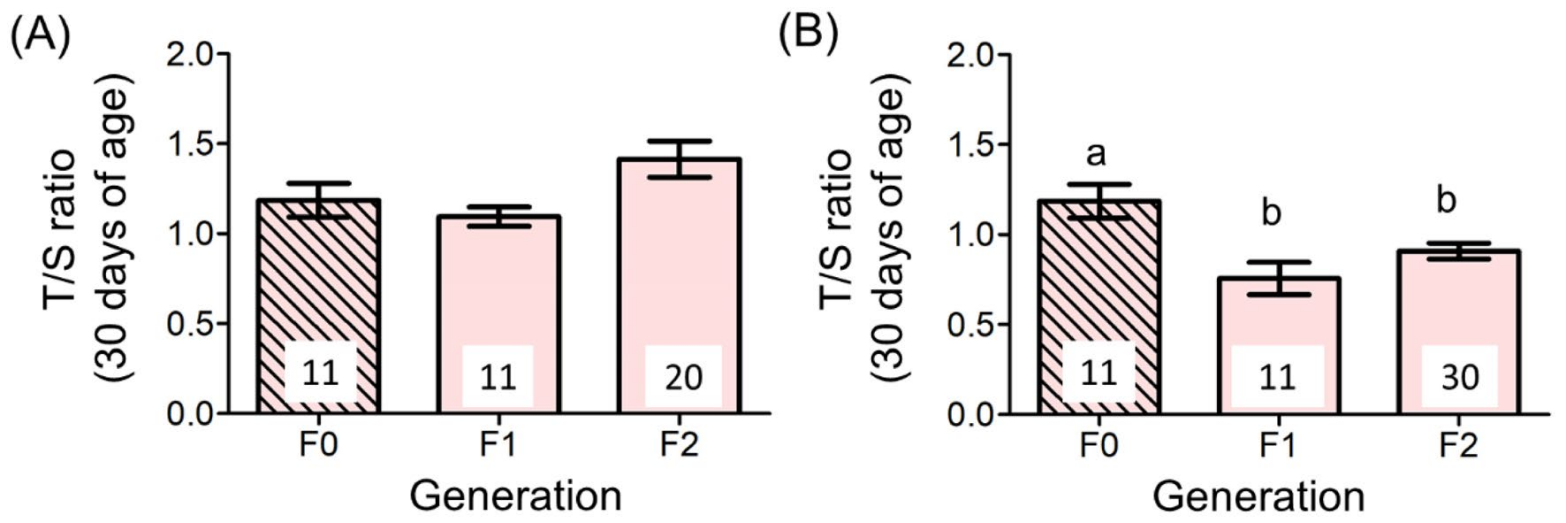
The negative effect of mother age at breeding on offspring telomere length persists across two generations. Thirty‐day telomere length (mean ± SE) in the 11 F0 females (hatched bar) who bred in young and old ages, and in the F1 and F2 females who came from the young (A) or old (B) grandmother line; numbers indicate sample sizes (one data point in (B) is missing in the F1 generation), different letters denote *p <* 0.05 (post hoc contrasts: F0 vs. F1: *p* = 0.0007; F0 vs. F2: *p =* 0.021; F1 vs. F2: *p* = 0.123).

F2 offspring of both sexes had substantially shorter telomeres when their mothers (F1) came from the breeding event that had taken place when their (F0) grandmothers were old (*p* < 0.0001; Figure 1A; Table 1). This effect of grandmother age was substantial (43% longer in the ‘young grandmother at breeding’ line; Figure 1A), consistent across broods (Figure 1B), and of a similar magnitude to the 36% reduction found in the F1 generation (Marasco et al. 2019). The other variables in the model were not significant (Table 1). The body mass of the F1 females at pairing and their subsequent breeding performance (clutch sizes and number of offspring (F2) reared up to fledging) did not differ depending on whether their mothers (F0 females) were bred as young or old adults (full statistics in Table 2a–c). We also found no effect of grandmother (F0) age at breeding on the body mass of grandoffspring (F2) at fledging (Table 2d). No decline in 30‐day telomere length across the three generations of females (F0, F1 and F2) was evident in the ‘young grandmother (F0) at breeding’ line (Figure 2A) but was clearly evident in the ‘old grandmother (F0) at breeding’ line (Figure 2B; Table 3).

**TABLE 1:** GLMM to examine the effect of grandmother age at reproduction (young or old) on telomere length (T/S ratio; values ln‐transformed) of grandoffspring (F2) at fledging (ca. 30 days of age). Values in bold indicate statistical significance (*p* < 0.05).

Parameter	Estimate	SE	*df*	*t*	*p*
F0 grandmother identity (*r*)	0.004 ± 0.060				
F1 mother identity (*r*)	0.007 ± 0.084				
Residual	0.082 ± 0.286				
Intercept	0.708	0.488	29.271	1.450	0.158
**Grandmother (F0) age at breeding [old]**	**−0.461**	**0.073**	**10.246**	**−6.349**	**< 0.0001**
Grandoffspring (F2) sex [male]	0.008	0.068	75.895	0.118	0.907
Grandoffspring (F2) hatching order	0.008	0.025	69.188	0.311	0.757
Clutch size (F2)	0.027	0.042	19.216	0.641	0.529
Grandoffspring (F2) fledging body mass	−0.040	0.033	36.976	−1.187	0.243

*Note:* Model output shows coefficient estimates for fixed factors (coefficients for categorical variables refer to the level shown in brakets); (*r*) indicates the random factor and its associated variance ± standard deviation.

**TABLE 2:** GLM/GLMM to examine the potential effect of age at reproduction (young or old) of F0 females on (A) the body mass at pairing of their daughters (F1), (B) the clutches these daughters produced, (C) the number of offspring (F2) they reared up to fledging (30 days of age) and (D) the fledging mass of the F2 offspring.

(A)					
Parameter	Estimate	SE	*df*	*t*	*p*
F0 mother identity (*r*)	0.615 ± 0.785				
Residual	3.905 ± 1.976				
Intercept	18.286	0.641	20.471	28.527	< 0.0001
Mother (F0) age at breeding [old]	−1.480	0.826	10.667	−1.791	0.102

(B)					
Parameter	Estimate	SE	*df*	*t*	*p*
F0 mother identity (*r*)	0.158 ± 0.397				
Residual	0.879 ± 0.938				
Intercept	5.091	0.307	20.528	16.582	< 0.0001
Mother (F0) age at breeding [old]	−0.205	0.392	12.337	−0.522	0.611

(C)				
Parameter	Estimate	SE	*t*	*p*
Intercept	3.636	0.305	11.930	< 0.0001
Mother (F0) age at breeding [old]	0.280	0.422	0.664	0.514

(D)					
Parameter	Estimate	SE	*df*	*t*	*p*
F1 mother identity (*r*)	0.589 ± 0.768				
Residual	0.596 ± 0.772				
Intercept	14.389	0.280	26.789	51.286	< 0.0001
Grandmother (F0) age at breeding [old]	−0.724	0.364	21.393	−1.989	0.060
Grandoffspring (F2) sex [male]	0.278	0.190	71.031	1.456	0.147

*Note:* Model outputs show coefficient estimates for fixed factors (coefficients for categorical variables refer to the level shown in parenthesis); (*r*) indicates the random factor and its associated variance ± standard deviation. In (C) due to low variance of the random factor, the output of a general linear model is shown instead; in (D) the identity of grandmothers (F0) could not be entered as an additional random factor due to low variance.

**TABLE 3:** GLMM to examine potential changes in average 30‐day (i.e. fledging) telomere length of females across the three generations (F0, F1 and F2). Values in bold indicate statistical significance (*p* < 0.05).

(A)					
Parameter	Estimate	SE	*df*	*t*	p
F0 grandmother identity (*r*)	0.008 ± 0.090				
Residual	0.059 ± 0.242				
Intercept	0.132	0.079	37.223	1.698	0.098
Generation [F1]	−0.055	0.103	30.370	−0.532	0.599
Generation [F2]	0.163	0.092	32.722	1.772	0.086

(B)					
Parameter	Estimate	SE	*df*	*t*	*p*
F0 grandmother identity (*r*)	0.017 ± 0.132				
Residual	0.080 ± 0.282				
Intercept	0.132	0.094	42.692	1.409	0.166
**Generation [F1]**	**−0.487**	**0.120**	**39.413**	**−4.045**	**0.0002**
**Generation [F2]**	**−0.283**	**0.101**	**41.586**	**−2.813**	**0.007**

*Note:* In (A) we modelled telomere length data from the F0 grandmothers, as well as their F1 daughters and F2 granddaughters that these F0 mothers produced when bred as young adults (‘grandmother young at breeding’ line); in (B) we modelled the telomere length data from the F0 grandmothers, as well as their F1 daughters and F2 granddaughters that these F0 females produced when bred as old adults (‘grandmother old at breeding’ line). Model outputs show coefficient estimates for fixed factors (coefficients for categorical variables refer to the level shown in brackets); (*r*) indicates the random factor and its associated variance ± standard deviation. Significant factors are highlighted in bold.

## 4 Discussion

F2 offspring sharing the same grandmother (F0) have substantially shorter telomeres when their mother (F1) came from the breeding event that took place when the grandmothers were old in comparison with those whose mothers came from their grandmother's young breeding event. The mothers (F1) of these F2 offspring were young at breeding, and there was no evidence that these mothers varied in condition. The body mass at breeding of the F1 females did not differ depending on the age at breeding (young or old) of their mothers (F0), nor did any aspect of their breeding performance or growth of their chicks (F2). Nor is the effect of old grandmother (F0) age at breeding on grandoffspring (F2) telomere length likely to be attributable to carry‐over effects of prior breeding events rather than actual chronological age at breeding *per se*. The grandmothers bred only four times over their lifespans (Marasco et al. 2018), and other studies have shown that the cost of reproduction for zebra finches rearing an unmanipulated brood size in captivity is very small, even for birds breeding several times per year (Heidinger et al. 2012).

The effects of grandmother age on grandoffspring telomere length shown here are in line with the predictions based on a germline‐transmitted effect of maternal age on offspring telomere length, with the F2 offspring inheriting telomeres that are in effect ‘older’, in cellular terms, than their chronological age. In both birds and mammals, oocytes are formed only during embryonic development, and oocyte telomere length and telomerase activity can decline with long‐term storage in the female ovary (Monaghan and Metcalfe 2019). It is likely, therefore, that the effect on telomeres has come about through oocyte deterioration in the old grandmothers, affecting all tissues of the resulting offspring. This could come about through a number of routes. Direct oxidative or other damage to the oocyte telomeres might have occurred, possibly linked to deterioration of mitochondrial function or changes in telomere maintenance in the oocytes or in the embryo. Reduced telomere length and reduced telomerase activity in old oocytes have been demonstrated in mice (Yamada‐Fukunaga et al. 2013) and shorter telomeres linked to oxidative damage in human oocytes from older women (Kordowitzki 2021; see also Monaghan and Metcalfe 2019 for discussion of oocytes deterioration in older females). It is of course also possible that the route is more complex, for example, a germ line mediated effect that influences telomere length re‐setting during embryogenesis, but such an effect is speculative. It is also interesting to note that the substantial effect of maternal age on offspring telomere length is evident even when the father is young, but little is known about how the maternally and paternally inherited telomere length contributes to the setting of offspring telomere length during embryonic development. In this study, the telomere lengths of both male and female grandoffspring were similarly affected by grandmaternal age at breeding suggesting that the multi‐generational transmission of shorter telomeres from the maternal line is not influenced by sex‐dependent pathways.

Long‐lived zebra finches (reaching 7 years of age) have on average ca. 33% longer rbc telomere length at fledging than short‐lived individuals (dying between 1 and 4 years of age) (Heidinger et al. 2012). Thus, the shortened telomeres we find at fledging in the grandoffspring that came from the ‘old grandmother at breeding’ line (on average of 43% shorter relative to the grandoffspring that came from the ‘young grandmother at breeding’ line) are likely give rise to a substantial reduction in their lifespan and hence in their reproductive value. This could have consequences for parental fitness and, where the effect is strong, would favour increased reproductive effort at younger ages. However, that in most birds and mammals both sexes reproduce throughout adult life, albeit with reduced success, suggests that some fitness benefits from breeding at older ages must still occur (Monaghan and Ivimey‐Cook 2023). For example, later‐life offspring might adjust their own reproductive investment to at least partially compensate for being born ‘older’, such as via faster maturation rates and earlier peaks in reproductive performance (Plaistow et al. 2015).

In zebra finches, we have also found that male age has adverse effects on offspring telomere length (Noguera, Metcalfe, and Monaghan 2018). All fathers used in this study were young, but it is clear that having a young father did not compensate for the effect of having an older mother and we do not know whether an old grandfather would have an additive effect to that of an old grandmother. It would also be interesting to know how these effects influence mate choice and strategic allocation of reproductive effort. Zebra finches are monogamous and form strong pair bonds, but re‐pairing and extra‐pair copulations do occur (Zann 1996). Males selectively pair with more fecund females (Monaghan, Metcalfe, and Houston 1996), so may be less likely to breed with old females, thereby avoiding producing offspring with reduced reproductive value. However, it is also possible that breeding and foraging experience of older partners has beneficial effects on offspring quality in the wild that are not evident in a captive situation. Further work would be needed to assess this. In our study, the F1 mothers were young at the time of breeding, thereby excluding effects due to F1 maternal ageing. However, it would also be very interesting to know whether the effects of grandmother age are compounded when maternal age is also old. Given the evidence of telomere shortening and reduced telomerase activity in oocytes during reproductive ageing (Yamada‐Fukunaga et al. 2013; Hao et al. 2023), it seems likely that female infertility would eventually occur once oocyte telomere length falls below a certain level and embryos are no longer viable. This could contribute to the evolution of a female post‐reproductive life stage (Monaghan and Ivimey‐Cook 2023).

To conclude, our findings demonstrate a hidden legacy that can be transmitted across generations and negatively influence offspring lifespan and reproductive value. This means that the reproductive value of offspring produced at different ages needs to be taken into account in demographic modelling and in the study of life‐history evolution. Evolutionary biologists and ecologists need to look beyond a single generation and current environmental circumstances to fully understand the causes of inter‐individual variation in ageing rates and age‐specific reproductive effort. Future intergenerational experiments monitoring the lifespan and reproductive performance of grandoffspring from old mothers and old fathers would be particularly valuable to improve our knowledge of processes underlying the evolution of ageing and within‐species diversity in life‐history strategies.

## Author Contributions

W.B., P.M. and V.M. designed the study; W.B., K.G., P.M. and V.M. performed the animal experiments; data curation: W.B., K.G. and V.M.; W.B., V.M. and S.R. performed the laboratory analyses; V.M. and P.M. analysed the data; V.M. and P.M. wrote the manuscript. All authors commented on and approved the final version.

## Conflicts of Interest

The authors declare no conflicts of interest.

### Peer Review

The peer review history for this article is available at https://www.webofscience.com/api/gateway/wos/peer‐review/10.1111/ele.70043.

## Data Availability

The data and code supporting the results are made available at http://datadryad.org/stash/share/C7XiSwP0r7zQmLKqpCLTPk4x_H7FuzCi1VuOqQfT098.
